# Mental health in the “era” of artificial intelligence: technostress and the perceived impact on anxiety and depressive disorders—an SEM analysis

**DOI:** 10.3389/fpsyg.2025.1600013

**Published:** 2025-06-02

**Authors:** Daniela-Elena Lițan

**Affiliations:** Department of Psychology, West University of Timișoara, Timișoara, Romania

**Keywords:** artificial intelligence, technostress, depression, anxiety, mental health

## Abstract

**Introduction:**

The aim of the current study is to analyze the relationship between mental health and the accelerated implementation and use of Artificial Intelligence (AI) in Romanian society. Given the growing integration of AI technologies, understanding their psychological impact is increasingly relevant.

**Methods:**

The perceived impact of the changes brought by AI technology was measured using the Technostress Creators Questionnaire. Mental health, assessed in terms of anxiety and depression disorders, was measured using the Depression Anxiety Stress Scales (DASS-21R). Data were analyzed using Structural Equation Modeling (SEM).

**Results:**

The results supported the proposed model and confirmed both tested hypotheses, indicating that anxiety and depression symptoms are significantly associated with AI-related technostress.

**Discussion/Conclusion:**

Due to the cross-sectional design, the findings should be interpreted as associative rather than causal. Nevertheless, this study provides an important contribution to the literature, addressing a notable gap in research regarding the psychological implications of AI adoption in society.

## 1 Introduction

Artificial Intelligence (AI) refers to the ability of a computer or machine to imitate the skills of the human mind, in the sense of learning from previous experiences, understanding and responding to language, decisions and problems (Thormundsson, 2024). AI can be found in various forms, such as natural language processing, robotics, neural networks and virtual assistants (Dang et al., 2025).

The benefits of AI integration into various applications are undeniable, significantly enhancing outcomes in domains such as medical diagnostics, autonomous vehicles, smart homes, social media, chatbots, virtual assistants, financial analysis (Pachegowda, 2023), and scientific research (França, 2023). However, its rapid and widespread adoption (Rodríguez, 2024) also carries the potential to generate or exacerbate mental health issues, particularly anxiety and depression. In this study, we refer to these manifestations as clinical disorders of anxiety and depression.

The root causes of such disorders can be explored both through existing literature and direct societal observation. AI-driven transformations affect all sectors (Gao and Wang, 2023) and often trigger psychological responses such as denial, shock, frustration, and anger (Bringselius, 2010), which may lead to anxiety (Ahmead et al., 2024; Robinson et al., 2013), followed by depression as individuals move through stages of adaptation: denial, anger, bargaining, depression, and acceptance (Sendrea, 2023).

Except for the human mind’s resistance to change, in the specialized literature we also find a series of research works analyzing how digital technology can negatively influence mental health (Abeliansky et al., 2024), as follows:

- digital burnout: having arisen as a consequence of the constant use of Internet and digital devices (Kardefelt-Winther, 2014), this syndrome manifests itself through physical, psychological and social problems, low levels of productivity, fatigue, inability to have control over emotions and inability to cope with routine (Erten and Özdemir, 2020);
- doomscrolling: according to the authors (Satici et al., 2023), doomscrolling can lead to higher levels of psychological distress and lower levels of mental well-being indicators (mental well-being, life satisfaction, and life harmony);
- the psychological effects of automation: according to research (Abeliansky et al., 2024), the adoption of industrial robots has significant negative effects on the employees’ mental health, and the effect is mediated by performance and concerns about job security.

In other words, if we are considering AI or another digital technology to which people can have unlimited access, taking into account the human mind “architecture,” the reactions mentioned above, amplified or not, depending on the context, would have a great potential for occurrence.

On the other hand, looking at today’s society and daily life, we can see people’s concerns about the fact that jobs, in the near future, could be replaced by AI – continuously improving in accuracy, robustness and coverage until replacing human experts (Gao and Wang, 2023) with robots or automation technologies (Abeliansky et al., 2024). However, the World Economic Forum announced, in January 2025, an increase of 78 million workplaces by 2030, taking into account the elimination of current jobs with repetitive activities and the creation of new workplaces in the context of the implementation of emerging technologies (AI, automation, extended digital access) and the green transition (World Economic Forum, 2025). Considering this topic, the Mental Health Europe organization highlighted in its paper (Mental Health Europe, 2022), in 2022, the potential risks brought to the workplace by the implementation of AI and digital technologies used to manage employees: creating an unsafe and competitive environment where the pressure felt can lead to anxiety, stress, low self-esteem, while AI tools used to monitor employees’ health can undermine their freedom and autonomy.

Given that it is a new technology, recently introduced into people’s lives, the specialized literature on the subject of AI and its consequences on mental health faces great deficiencies, the number of studies being very low. However, in the works identified on this topic, we can notice the researchers’ interest in analyzing: the occurrence of anxiety disorders (Alkhalifah et al., 2024; Rodríguez, 2024) and depression (Xu et al., 2023), life satisfaction (Hinks, 2024), self-esteem, psychological well-being (Salah et al., 2024).

The AI technology is not like any other technology, given the speed of task execution, power, versatility, and the extent to which it blurs the distinction between man and machine (Rodríguez, 2024). In this context, the studies existing in the specialized literature, conducted on other types of technologies in relation to mental health, in the absence of those focusing on AI, can provide only a direction regarding the potential consequences, and not the scientifically validated results on the basis of which decision-makers can take measures to support the population and responsibly implement AI (Alkhalifah et al., 2024; Nussberger et al., 2022).

Research in the field of digital stress suggests that anxiety may arise from individuals’ exposure to fast-evolving technology, with AI as a prominent case. The feelings of uncertainty, lack of control, and cognitive overload triggered by continuous AI integration may facilitate the development of anxiety and/or intensify pre-existing symptoms (Cengiz and Peker, 2025; Kim et al., 2025; Li and Huang, 2020). Recent findings show that technostress correlates with higher levels of psychological tension and emotional instability, with AI tools acting as both productivity enhancers and anxiety amplifiers (Chuang et al., 2025; Stănescu and Romașcanu, 2024).

Therefore, we formulate the following first hypothesis:

*H1*: The technostress perceived as a result of the accelerated pace of AI implementation and use is positively associated with anxiety.

In parallel, several studies emphasize that long-term exposure to AI-driven work environments, job insecurity due to automation, and constant digital monitoring are significantly associated with emotional exhaustion, sadness, and depressive symptoms (Xu et al., 2023; Zheng and Zhang, 2025). The loss of human agency, algorithmic bias, and perceived lack of control can lead individuals toward cognitive withdrawal and helplessness (Ahmad et al., 2023; Uygungil-Erdogan et al., 2025), conditions which are fertile ground for depressive disorders.

Therefore, the second hypothesis of the study is:

*H2*: The technostress perceived as a result of the accelerated pace of AI implementation and use is positively associated with depression.

The conceptual model proposed in this study is informed by and aligned with recent empirical and theoretical work addressing AI-induced technostress and its impact on mental health (Ahmad et al., 2023; Cengiz and Peker, 2025; Chuang et al., 2025; Kim et al., 2025; Stănescu and Romașcanu, 2024; Uygungil-Erdogan et al., 2025; Xu et al., 2023; Zheng and Zhang, 2025). These studies served as a valuable foundation for the development of the model presented below.

**Figure 1:**
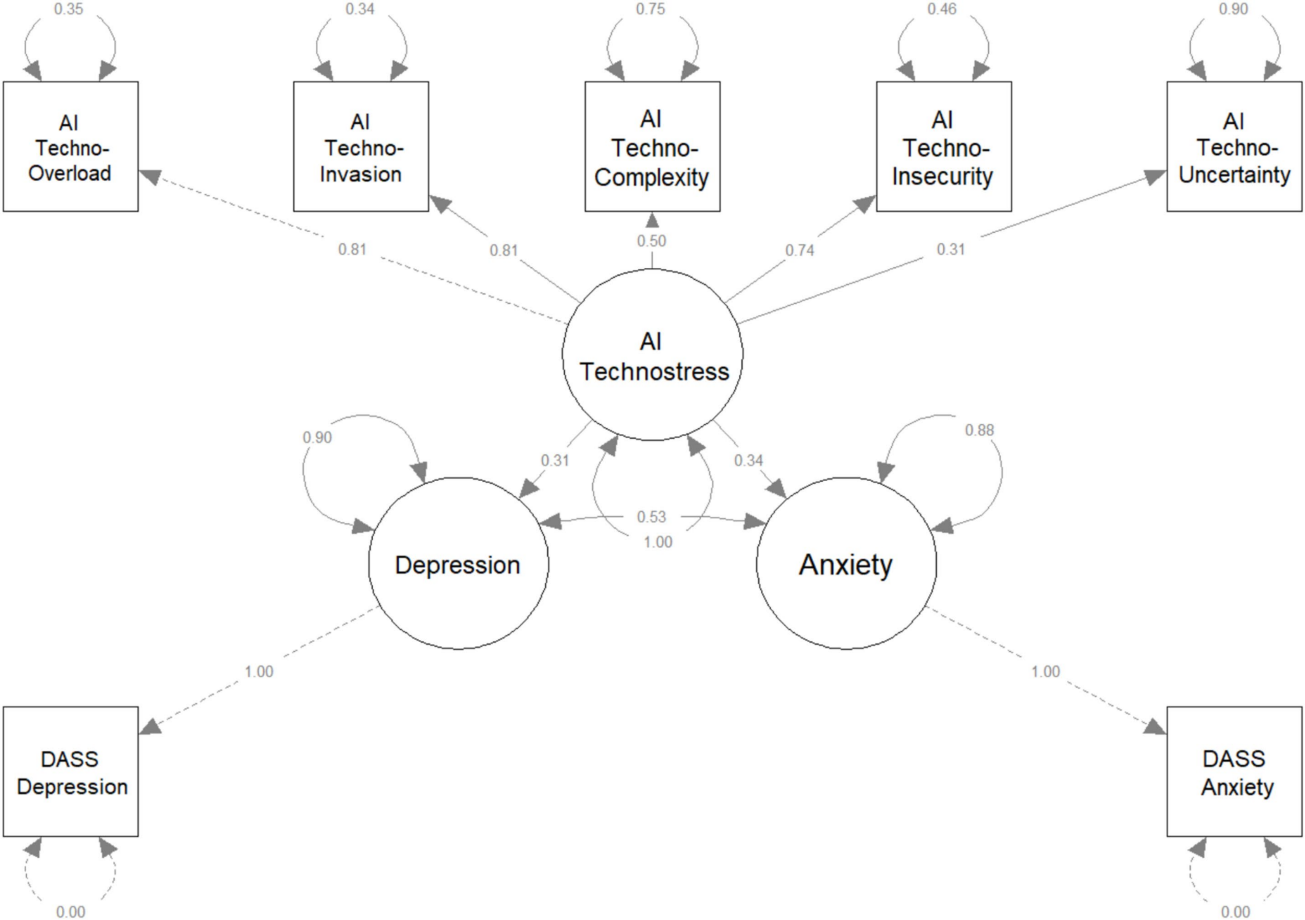
The hypothesized model.

The theoretical model that underlies these assumptions is illustrated in Figure 1.

The scale used in the current research is the Technostress creators scale (Ragu-Nathan et al., 2008) which measures the level of stress induced by technology, and in the context of AI, it can assess the impact of automation and interaction with AI systems. The depression and anxiety scales from the DASS-21R questionnaire (Lovibond and Lovibond, 1995) were also used to assess depression and anxiety disorders.

The novelty of this study lies in its targeted focus on the Romanian adult population, addressing a gap in the literature concerning AI-induced technostress in general society, beyond employees or students. Furthermore, we aim to contribute empirically to a field where theoretical assumptions often surpass validated data.

The remainder of this paper is structured as follows: Section 2 presents the methodology, including the study design (2.1), participants (2.2), measures (2.3), procedure (2.4), and statistical analysis approach (2.5). Section 3 reports the results of the SEM analysis. Section 4 provides a discussion of the main findings and their implications. Finally, Section 5 outlines the study’s limitations and proposes future research directions, including longitudinal investigations and cross-cultural comparisons related to AI-induced technostress.

We need to mention the fact that this study does not aim to build a global model of depression and anxiety, but to specifically measure the impact of technostress generated by the implementation of AI, at the level of the adult population, Romanian citizens.

## 2 Method

### 2.1 Study design

The current study was approved by the Scientific Council of University Research and Creation from the West University of Timișoara, Romania, in August 2024 (process number: 53165/02.08.2024) and was conducted in accordance with the World Medical Association Helsinki declaration. The participants in the study were informed about the context, objective and purpose of the study and all provided informed consent.

The current research has a cross-sectional survey design and the responses to the questionnaires used were collected online, using the Google Forms platform, between 11.12.2024 and 08.02.2025. The choice of a cross-sectional design with online data collection was made to ensure efficiency, wide reach, and cost-effectiveness, especially given the study’s target population and exploratory nature.

### 2.2 Participants

The selection criteria for the participants’ inclusion in the study were: Romanian citizenship, age between 18 and 65 years, female or male, and the ability to understand a written text.

In the current study, 217 people (82 men, 135 women), adult Romanian citizens, aged 18 to 62 years (*M* = 36.15, SD = 11.92) participated voluntarily, out of whom: 143 employees, 40 students, 27 freelancers, 3 unemployed, 2 retired people (pensioners), 2 housewives. Other demographic characteristics of the study participant group:

- level of education (last school graduated): high school: 52 participants, college: 78 participants, Master’s Degree: 76 participants, Doctorate/post-doctorate: 11 participants;
- area of residence: 174 people live in urban areas, while 43 people live in rural areas;
- marital status: single: 64 people, in a relationship: 153 people.

A detailed overview of the participants’ demographic data is provided in Table 1.

**Table 1:** Demographic characteristics of the participants (*N* = 217).

Variable	Category	Frequency (N)	Percentage (%)
Gender	Male	82	37.8
	Female	135	62.2
Age	Mean = 36.15, SD = 11.92	–	–
Occupation	Employee	143	65.9
	Student	40	18.4
	Freelancer	27	12.4
	Unemployed	3	1.4
	Retired	2	0.9
	Housewife	2	0.9
Education level	High school	52	24.
	College	78	35.9
	Master’s degree	76	35
	PhD/Postdoc	11	5.1
Area of residence	Urban	174	80.2
	Rural	43	19.8
Marital status	Single	64	29.5
	In a relationship	153	70.5

Age is reported as Mean and Standard Deviation. Percentages are rounded to one decimal place.

Conditions for excluding the responses received from participants: the participant’s refusal to give their consent to participate in the study – the consent to participate in the study being the first condition to be met, before the study questions were displayed on the screen (there was 1 case, which was excluded from the research) and abandoning the questionnaire once started and not completing it in full (no such situations were found in the database).

The sample size was calculated *a priori* with the G*Power program, which for an average effect, a power of 0.80, a type I error equal to 0.05 and one predictor displayed a minimum size of 55 participants.

### 2.3 Measures

#### 2.3.1 The technostress scale (Technostress creators)

Technostress is, in fact, the stress that users experience as a result of using information systems and technologies (Costin and Ona, 2023). The technostress scale as found in the work (Ragu-Nathan et al., 2008) is made up of two components: Technostress creators (represents the stress-generating factors in relation to technology) and Technostress inhibitors (describes the organizational mechanisms that have the potential to reduce the effects of technostress).

In the current study, the Technostress creators component used measures the level of the stress perceived as a result of using AI. This scale has also been used in other studies with Romanian participants, with excellent reliability: (Cazan et al., 2024; Truța et al., 2023).

The Technostress creators scale is made up of 5 factors, which according to the authors (Ragu-Nathan et al., 2008), can be viewed as different aspects or dimensions of technostress:

- Techno-overload: this factor describes situations where technology (AI) forces users to work faster and harder.
- Techno-invasion: this factor describes situations in which the connection with technology (AI) is permanent, regardless of the personal or professional context.
- Techno-complexity: this factor describes situations where people feel compelled to spend time and effort to learn and understand technology (AI).
- Techno-insecurity: this factor refers to situations where people feel threatened by losing their workplaces, either because of technology (AI) taking over human activities, automating them, or because of other people who have better skills in using technology (AI).
- Techno-uncertainty: this factor describes contexts in which continuous changes in technology (AI) create uncertainty and lack of stability, leading to a permanent education and adaptation of people.

The Technostress creators questionnaire allows participants to record responses on a 5-option Likert scale (from 1-Totally Disagree, to 5-Totally Agree).

The 5 factors of the Technostress creators questionnaire, used in the current research, had a good internal consistency, with Cronbach’s alpha values: 0.881 (Techno-overload), 0.810 (Techno-invasion), 0.847 (Techno-complexity), 0.830 (Techno-insecurity), 0.907 (Techno-uncertainty).

While this scale was not specifically designed for AI technologies, its dimensions have been contextually interpreted to reflect AI-related stress factors. The Technostress Creators scale was therefore selected due to its multidimensional structure, empirical use in digital stress research, and its adaptability to new technological contexts. Although originally developed for general technologies, its factors remain conceptually relevant for capturing stressors generated by AI systems.

#### 2.3.2 The DASS-21R questionnaire (anxiety, depression and stress assessment scales)

The levels of depression and anxiety experienced by the study participants in relation to AI technology were assessed using the DASS-21R questionnaire (Lovibond and Lovibond, 1995): anxiety and depression assessment scales.

The DASS-21R questionnaire in Romanian, adapted and calibrated for the Romanian population (Perţe and Albu, 2011), includes 21 items, divided into three scales: anxiety, depression and stress. According to the Romanian manual (Perţe and Albu, 2011), the questionnaire was built with the aim of measuring the constructs (anxiety, depression and stress) by identifying the essential features of each syndrome and eliminating the overlap of items between scales. The DASS-21R questionnaire assesses the emotional state in relation to situations which are also outside the testing context (not only from the moment of testing) and can be used both in research and in a clinical context.

The DASS-21R questionnaire allows participants to record responses on a 4-option Likert scale (from 0 - did not suit me, to 3 - suited me very much or almost all the time).

The 2 scales of the DASS-21R questionnaire, used in the current research, had good internal consistency, with Cronbach’s alpha values: 0.867 (Anxiety), 0.869 (Depression).

The DASS-21R questionnaire was used because it is brief, psychometrically robust, and widely employed in both clinical and research settings. Its Romanian adaptation ensures cultural validity and consistency in measuring depression and anxiety symptoms in the target population.

### 2.4 Procedure

The collection of responses was carried out online, through the Google Forms platform, from 11.12.2024 to 08.02.2025. The questions were addressed to the participants in Romanian. The questionnaire link was shared on professional, social, mobile messaging platforms, and sent by email to the target groups. The questionnaire was completed by the participants voluntarily, without compensation and anonymously. Informed consent regarding participation in the study was requested and received from the participants, after they were informed about the objectives of the research and also that they could also withdraw at any time during the study.

The general questionnaire was composed of demographic questions (year of birth, gender, level of education, professional status, marital status) followed by the Technostress questionnaire (Technostress creators) and the DASS-21R scales for assessing anxiety and depression.

The study was pre-registered on the Open Science Framework platform (objectives, main hypotheses, study design, data collection procedure, measured variables and statistical analysis plan), before data collection. The pre-registration can be consulted at: https://osf.io/yefdk/?view_only=728f65c5ffbd43ab9818aaaff35abc35 (the current study is a sub-study of the research topic: Digital transformation as a factor in the manifestation of clinical symptoms of depression and anxiety, mediated by the level of self-esteem).

### 2.5 Statistical analysis

In order to analyze the model proposed in this research (see Figure 1), Structural Equation Modeling (SEM) was used. In this respect, the RStudio software product–version 2024.12.0 (build 467)–Windows 10 Pro operating system was used. The packages used in order to perform the statistical analysis and graphical representation of the model were: lavaan and semPlot.

SEM is a multivariate statistical method used to model complex relationships between directly observed variables and latent (indirect) variables. It involves estimating parameters for a system of simultaneous equations (Stein et al., 2012), allowing the analysis of interdependencies between variables and the testing of theoretical hypotheses.

In order to evaluate the proposed model (data-model fit), the criteria established in the paper (Hu and Bentler, 1999) were used, more precisely: Comparative Fit Index (CFI) ≥ 0.96, Standardized Root Mean Square Residual (SRMR) ≤ 1.0, or Root Mean Square Error of Approximation (RMSEA) ≤ 0.06 and Standardized Root Mean Square Residual (SRMR) ≤ 0.08.

Although common method bias is a potential concern in studies using self-report instruments in a single session, the use of SEM with multiple latent constructs and the assessment of model fit through standard indices (Hu and Bentler, 1999) provide a robust approach to evaluating and mitigating such bias.

## 3 Results

According to the criteria (Hu and Bentler, 1999), the test indicated a good fit of the model, as follows: CFI = 0.988, SRMR = 0.040, RMSEA = 0.043, 90%CI = [0.000, 0.085]. Also, CMINF/DF = 1.39, NFI = 0.959, *p*-value (Chi-square) = 0.151, df = 21, TLI = 0.98.

The descriptive statistics and correlations between study variables are presented in Table 2. According to Table 2, both the manifestation of anxiety disorders and the manifestation of depression disorder are positively correlated with technostress factors (induced by AI), implicitly supporting the two hypotheses of the study, H1 and H2. The exception is the Techno-uncertainty factor for which there is no correlation for either type of disorder. Anxiety disorders are positively correlated with technostress factors, as follows: Techno-overload (*r* = 0.267, *p* < 0.001), Techno-invasion (*r* = 0.298, *p* < 0.001), Techno-complexity (*r* = 0.234, *p* < 0.001), Techno-insecurity (*r* = 0.233, *p* < 0.001). The depressive disorder is positively correlated with technostress factors, as follows: Techno-overload (*r* = 0.206, *p* < 0.01), Techno-invasion (*r* = 0.267, *p* < 0.001), Techno-complexity (*r* = 0.238, *p* < 0.001), Techno-insecurity (*r* = 0.244, *p* < 0.001).

**Table 2:** Descriptive statistics and correlations between the investigated variables.

Variable	Mean	SD	1	2	3	4	5	6	7	8	9
AI - Techno-overload	12.922	5.501	—								
AI - Techno-invasion	5.502	3.022	0.673***	—							
AI - Techno-complexity	11.475	4.772	0.391***	0.383***	—						
AI - Techno-insecurity	9.29	4.243	0.577***	0.589***	0.425***	—					
AI - Techno-uncertainty	11.336	4.808	0.288***	0.208**	0.091	0.304***	—				
Anxiety	5.111	4.597	0.267***	0.298***	0.234***	0.233***	0.004	—			
Depression	3.848	4.257	0.206**	0.267***	0.238***	0.244***	0.043	0.58***	—		
Gender			−0.023	0.02	0.225***	0.024	0.001	0.189**	0.039	—	
Age	36.152	11.92	−0.116	−0.182**	0.173*	−0.183**	−0.084	−0.072	−0.206**	0.334***	—

*p < 0.05, **p < 0.01, ***p < 0.001; SD = Standard Deviation, Gender is coded as 0 = male, 1 = female. Age is expressed in years. Variables: 1 = AI–Techno-overload, 2 = AI–Techno-invasion, 3 = AI–Techno-complexity, 4 = AI–Techno-insecurity, 5 = AI–Techno-uncertainty, 6 = Anxiety, 7 = Depression, 8 = Gender, 9 = Age.

The technostress perceived as a result of the implementation of AI is included in the model as a latent variable, having as measures its five factors: Techno-overload, Techno-invasion, Techno-complexity, Techno-insecurity and Techno-uncertainty. The values of the loadings obtained are as follows:

- Techno-overload: 0.809 (factor strongly related to technostress (AI));
- Techno-invasion: 0.813 (factor strongly related to technostress (AI));
- Techno-complexity: 0.503 (factor moderately related to technostress (AI));
- Techno-insecurity: 0.735 (factor strongly related to technostress (AI));
- Techno-uncertainty: 0.314 (factor weakly related to technostress (AI)).

Therefore, as we can see above, the factors Techno-overload and Techno-invasion are the most important factors for technostress (AI), while the factor Techno-uncertainty has a weaker association with technostress (AI), suggesting that it may not be a strong predictor.

In their turn, the observed variables, anxiety disorders and depression, were measured using the DASS-21R questionnaire.

The analysis of the relationship between technostress (AI) and the symptoms of anxiety and depression highlighted the fact that technostress is a significant predictor for anxiety and depression disorders, and the effect on anxiety (*β* = 0.342) is slightly higher than on depression (*β* = 0.308), for *p* < 0.001 (for both relationships), which means that the effects are statistically significant. Also, the SEM analysis of the model highlighted the fact that technostress explains 11.7% of the variability of anxiety and 9.5% of the variability of depression. Therefore, we can conclude that the two other hypotheses of the current study were confirmed by the statistical analysis performed (see also Figure 1):

- H1: The technostress perceived as a result of the accelerated pace of AI implementation and use is positively associated with anxiety (*β* = 0.342, *p* < 0.001, R2 = 0.117).
- H2: The technostress perceived as a result of the accelerated pace of AI implementation and use is positively associated with depression (*β* = 0.308, *p* < 0.001, R2 = 0.095).

A summary of hypothesis testing results based on the SEM analysis is provided in Table 3.

**Table 3:** Hypothesis testing summary.

Hypothesis	Structural path	*β*	*p*-value	*R*^2^	Result
H1	Technostress (AI) → Anxiety	0.342	< 0.001	0.117	Supported
H2	Technostress (AI) → Depression	0.308	< 0.001	0.095	Supported

*p*-values reported for two-tailed tests; *β* = standardized regression coefficient; *R*^2^ = variance explained.

It is necessary to emphasize that SEM modeling was used to isolate the contribution of technostress to the manifestation of depression and anxiety symptoms, without introducing other factors that could distort this effect. This approach allows us to directly understand the relationship between AI and mental health, without overestimating the explanatory power of the model through additional predictors.

Also, as expected and as can be seen in Figure 1, anxiety and depression disorders are moderately correlated (depression-anxiety covariance: Std. all = 0.53, *p* < 0.001), suggesting that people with high levels of anxiety have an increased risk of depression and vice versa. This aspect is also confirmed by the specialized literature: Vasugi and Hassan (2019), Hoying et al. (2020), and Lu et al. (2023).

## 4 Discussion

The current research is, in fact, a natural extension of the existing studies in the specialized literature on the analysis of the relationship between technostress induced by the intensive use of technology and mental health, in the current study, the orientation of technology being toward the AI branch–a field that is still insufficiently researched.

The aim of the current study was to analyze the relationship between technostress resulting from the accelerated implementation of AI and mental health, on a sample of 217 adult Romanian citizens. We assumed that technostress (AI) positively influences the manifestation of anxiety and depression symptoms, starting from the specialized literature, as we showed in the first part of this paper, where we find, on the one hand, studies presenting the effects of digital technology on mental health, and on the other hand, resistance to change, as an emergent feature of the human mind, manifested as a reaction of the limbic system to “threats” and the unknown.

The descriptive statistical analysis showed that there were significant, positive correlations, with values around the average, between technostress (AI) factors – except for the techno-uncertainty factor, and anxiety and depression disorders, as presented in paragraph 3. Results of this paper, also in Table 2.

- Techno-overload positively correlates with anxiety (*r* = 0.267, *p* < 0.001) and depression (*r* = 0.206, *p* < 0.001), suggesting that people who feel overwhelmed by technology may have higher levels of emotional stress.
- Techno-invasion has a stronger link with anxiety (*r* = 0.298, *p* < 0.001) and depression (*r* = 0.267, *p* < 0.001), which shows that when technology invades personal life, the negative effects on mental health are significant.
- The moderate correlations: Techno-complexity–anxiety (*r* = 0.234, *p* < 0.001) and Techno-complexity–depression (*r* = 0.238, *p* < 0.001), highlight the difficulty perceived in using AI technology, which can induce feelings of incompetence and cognitive stress, contributing to an increased level of anxiety and depression.
- The moderate correlations: Techno-insecurity–anxiety (*r* = 0.233, *p* < 0.001) and Techno-insecurity - depression (*r* = 0.244, *p* < 0.001), highlight the fear that technology will replace human skills (for example, it may lead to job losses), and this fear can generate anxiety and depression.

We can also infer from Table 2 that young people feel more affected by technology in their personal lives than older people (correlation: Techno-invasion-age: *r* = −0.182, *p* < 0.01) and have more fears that technology could affect their professional development (correlation: Techno-insecurity-age: *r* = −0.183, *p* < 0.01), a result confirmed by the specialized literature (James and Sahu, 2023). As far as the complexity of technology (AI) in relation to gender is concerned, we can see that women, compared to men, have more fears related to the fact that they could be replaced or that they cannot adapt to the new AI technology (*r* = 0.225, *p* < 0.001). This finding regarding women’s reporting of technostress is consistent with previous findings in the literature (Estrada-Muñoz et al., 2021; Kasemy et al., 2022).

The two hypotheses of the analyzed model, which aimed to test the relationships between technostress generated by the accelerated implementation of AI technology in society and the manifestation of anxiety (H1) and depression (H2) symptoms, were confirmed. The regressions between technostress (AI) and anxiety disorders (H1: *β* = 0.342, *p* < 0.001, R2 = 0.117), and depressive disorder (H2: *β* = 0.308, *p* < 0.001, R2 = 0.095) are both significant, highlighting the fact that technostress (AI) is a significant predictor for the two types of disorders, and the effect on anxiety (*β* = 0.342) is slightly greater than on depression (*β* = 0.308).

The technostress (AI) within the model is well represented by its 5 factors, all of them having standardized loadings ranging from 0.314, *p* < 0.001 (Techno-uncertainty factor) to 0.813, *p* < 0.001 (Techno-invasion factor). Within the aforementioned range, we find the factors: Techno-overload (0.809, *p* < 0.001), Techno-insecurity (0.735, *p* < 0.001) and Techno-complexity (0.503, *p* < 0.001).

The influence of the Techno-overload factor, in the context of AI, on the manifestation of anxiety and depression symptoms, can be interpreted by the fact that the large volume of information and tasks managed by a person can lead to increased levels of anxiety and depression, given the excessive technological demands - a situation also mentioned in the works (Galvin et al., 2022; Vallone et al., 2023). The influence of the Techno-invasion factor on the manifestation of anxiety and depression symptoms can be interpreted by the fact that the penetration of AI technology into personal life, causing individuals to be constantly available and blurring the “boundaries” between professional and personal life, or just interacting with technology in situations where, until recently, the interaction was carried out with a human being (for example: customer service and CallCenter, retail, transportation, deliveries, education, etc.), can lead to emotional exhaustion, anxiety and depression. The current results are consistent with previous results, with reference to technology in general: (Smoroň and Schraggeova, 2022; Wu et al., 2017; Wu et al., 2020).

The fear that current human skills may be surpassed by the rapid advance of AI or the fear of not being able to keep up with the evolution of AI technology, in other words, Techno-insecurity can affect professional stability and even career, after years of training and professional experience, altering mental health (Yang et al., 2025), through the appearance of symptoms of anxiety and depression.

In their turn, the Techno-complexity and Techno-uncertainty factors, although having lower loadings than the first three factors of techno-stress (AI) mentioned above, can still cause anxiety and depression disorders, given that, on the one hand, people must learn to adapt to the new AI technology, overcoming the difficulties of understanding and using it, and on the other hand, continuous technological changes in the field of AI and the way they affect professional roles and responsibilities generate insecurity and uncertainty. Also, in support of the aforementioned, the research (Mougha et al., 2023) highlights the fact that exposing people to Techno-complexity and Techno-uncertainty generates exhaustion. Constantly maintaining a state of exhaustion (burnout) is in fact a risk factor for the installation of symptoms of anxiety and depression (Koutsimani et al., 2019).

The results of this study conducted on adult Romanian citizens are in line with the specialized literature that includes studies close to the current topic. For example, in the paper (Alkhalifah et al., 2024) the authors identified a high prevalence of existential anxieties related to the rapid advances of AI: from the fear of death, the unpredictability of fate, a sense of emptiness, anxiety related to the lack of meaning, guilt over potential catastrophes related to AI, to the fear of being blamed due to ethical dilemmas. In their turn, the authors of the paper (Xu et al., 2023) concluded that employees who perceive AI as a threat to their career are more prone to depression. In addition, the emotional exhaustion mediates this relationship, that is the fear of losing their job and technological stress lead to exhaustion, which, in its turn, increases the risk of depressive disorder.

Regarding the values of the coefficients of determination, obtained from the statistical analysis of the model presented above, R2 = 0.117–anxiety disorders and R2 = 0.095–depressive disorder, the low to moderate values are not surprising, since anxiety and depressive disorders are generally influenced by numerous factors external to the model (e.g., lifestyle, social support, genetic factors, etc.). However, our goal was not to explain the entire variance of these disorders, but to quantify the specific effect of technostress caused by AI. Also, in support of what has already been mentioned, we should notice that, in the specialized literature, in studies on the relationship between technology and mental health, predictive models often have a moderate explanatory power, for example the studies (Hashemi et al., 2022; Sommantico et al., 2023).

These findings also contribute to the theoretical understanding of technostress in the context of AI. The study confirms that AI-induced technostress can be viewed as a multidimensional construct relevant not only to workplace environments, but also to broader societal contexts in which individuals interact with AI technologies on a daily basis. By validating its association with anxiety and depressive symptoms, the results support theoretical models that integrate digital stressors into mental health frameworks and highlight the need to expand these models to include AI-specific challenges.

In practical terms, this study has implications for multiple stakeholders. For employees, increased awareness of AI-related stress and its potential mental health consequences can promote early coping strategies and healthier digital habits. For managers, the results suggest the importance of offering training programs that reduce techno-complexity and insecurity, and ensuring organizational cultures that respect psychological boundaries in AI use. At the level of companies and industries, these findings call for responsible AI integration policies that prioritize mental health, transparency, and human-centered implementation.

## 5 Limitations and future research directions

AI enables the development of invaluable services and takes part in more and more aspects of our lives (UNESCO, 2025). The way we approach AI will define the world we live in in the future (European Commission, 2025). These are just a few examples, in which important international institutions emphasize the importance of AI technology and the historical moment we are experiencing. In this context, a deep understanding of the dynamics between the impact of AI technology on mental health becomes essential.

Although the current research is descriptive, exploratory, differential, and correlational and provides valuable information, being one of the few studies in the specialized literature conducted on this topic (technostress generated by the accelerated implementation and use of AI in relation to mental health), it also has a number of limitations. As already mentioned previously, this study focused exclusively on the relationship between technostress and mental health, without including other predictors that could contribute to depression or anxiety. This decision was made to maintain the clarity of the analysis and to avoid introducing variables that are not directly relevant to the research objective.

An important limitation of the current study is its cross-sectional design, which precludes the ability to make a causal inference (Yue, 2023). Consequently, future studies should adopt a longitudinal design for a more nuanced understanding of the relationship (Bui and Duong, 2024): technostress (AI) – mental health impairment through the development of anxiety and depression symptoms over time.

The second limitation is the use of a technostress scale originally developed for general technologies. Although adapted for the AI context, the absence of a validated AI-specific scale highlights the need for future development in this area.

The third limitation is that the study focused on the general implementation and use of AI technology and was not specific to any application or field of activity (Chu et al., 2021). Therefore, future studies, for a “deeper” understanding of the topic of technostress (AI) – mental health, will need to conduct more targeted analyses in specific usage domains and technological contexts. Additionally, the sample consisted exclusively of Romanian citizens, which may limit the generalizability of the findings. Cultural and societal differences in the perception and adoption of AI technologies may influence the results, and future studies should replicate this model in other national or cultural contexts.

An important limitation, the fourth, is that the participants’ responses to the questionnaires used for this research were self-reported, a situation that could have led to the amplification or underestimation of the relationships between variables (Podsakoff et al., 2003).

The conceptual model highlights direct associations between AI-induced technostress and symptoms of anxiety and depression but does not incorporate potential mediation effects. This decision was intentional, aiming to maintain a parsimonious model in an emerging research area. Future studies should explore more complex mechanisms, including mediation and moderated mediation effects.

In conclusion, although the current research lays the groundwork for analyzing the relationship between AI-induced technostress and mental health, future studies should address the previously discussed limitations to gain a more nuanced understanding of this dynamic.

## Data Availability

The raw data supporting the conclusions of this article will be made available by the authors, without undue reservation.
